# Web-Based Alcohol, Smoking, and Substance Involvement Screening Test Results for the General Spanish Population: Cross-Sectional Study

**DOI:** 10.2196/jmir.7121

**Published:** 2018-02-16

**Authors:** Juan A Lopez-Rodriguez, Gabriel Rubio Valladolid

**Affiliations:** ^1^ Primary Care Research Unit Gerencia Asistencial de Atención Primaria Servicio Madrileño de Salud Madrid Spain; ^2^ General Ricardos Primary Health Care Centre Madrid Spain; ^3^ School of Health Sciences King Juan Carlos University Alcorcón, Madrid Spain; ^4^ Red de Investigación en Servicios de Salud en Enfermedades Crónicas (REDISSEC) Carlos III Health Institute Madrid Spain; ^5^ School of Medicine Complutense University of Madrid Madrid Spain; ^6^ Department of Psychiatry 12 Octubre University Hospital Madrid Spain; ^7^ Area 8, Neurociencias y Salud Mental Madrid Spain; ^8^ Red de Trastornos Adictivos (RTA) Carlos III Health Institute Madrid Spain

**Keywords:** screening, substance-related disorders, Web-based systems, primary health care, ASSIST

## Abstract

**Background:**

Information technology in health sciences could be a screening tool of great potential and has been shown to be effective in identifying single-drug users at risk. Although there are many published tests for single-drug screening, there is a gap for concomitant drug use screening in general population. The ASSIST (Alcohol, Smoking and Substance Involvement Screening Test) website was launched on February 2015 in Madrid, Spain, as a tool to identify those at risk.

**Objective:**

The aim of this study was to describe the use of a tool and to analyze profiles of drug users, their consumption patterns, and associated factors.

**Methods:**

Government- and press-released launching of a Spanish-validated ASSIST test from the World Health Organization (WHO) was used for voluntary Web-based screening of people with drug-related problems. The tests completed in the first 6 months were analyzed *.*

**Results:**

A total of 1657 visitors of the 15,867 visits (1657/15,867, 10.44%) completed the whole Web-based screening over a 6-month period. The users had an average age of 37.4 years, and 78.87% (1307/1657) screened positive for at least one of the 9 drugs tested. The drugs with higher prevalence were tobacco (840/1657, 50.69%), alcohol (437/1657, 26.37%), cannabis (361/1657, 21.79%), and sedatives or hypnotics (192/1657, 11.59%). Polyconsumption or concomitant drug use was stated by 31.80% (527/1657) of the users. Male respondents had a higher risk of having alcohol problems (odds ratio, OR 1.55, 95% CI 1.18-2.04; *P*=.002) and double the risk for cannabis problems (OR 2.07, 95% CI 1.46-2.92; *P*<.001). Growing age increased by 3 times the risk of developing alcohol problems for people aged between 45 and 65 years (OR 3.01, 95% CI 1.89-4.79; *P*<.001).

**Conclusions:**

A Web-based screening test could be useful to detect people at risk. The drug-related problem rates detected by the study are consistent with the current literature. This tool could be useful for users, who use information technology on a daily basis, not seeking medical attention.

## Introduction

The consumption of drugs and addiction are common problems throughout the world and a foreseeable cause of death [1]. In 2014, almost 250 million people between the ages of 15 and 64 years had consumed an illegal substance at least once. The number of adults addicted to a drug, in the world reached 29 million, according to the United Nations Office of Drugs and Crime [2].

The magnitude of this problem is quantified in Spain by the EDADES (Encuesta De Alcohol y Drogas en ESpaña)[3] survey, carried out twice a year by means of a home-based, door-to-door questionnaire addressed to a large part of the Spanish population. Its purpose is to determine the pattern of drug consumption among the Spanish population, engaging approximately 20,000 people.

The screening of problems related to the consumption of drugs such as alcohol and tobacco from a health perspective is a grade B evidence recommendation (meaning the Agency recommends the service to offer or to provide the service) both in the United States (United States Preventive Services Task Force) [4] as well as in Spain (Programa de Actividades Preventivas y Promoción de la Salud en Atención Primaria) [5]. However, the evidence accumulated to date for the simultaneous screening of several most common substances found in primary care or the general population remains scarce [6].

Tools have therefore been designed for screening subjects with addiction to substances, however, these are limited in detecting at-risk people who do not meet substance-related disorder criteria [7-10]. In 1997, the World Health Organization (WHO) developed ASSIST (Alcohol, Smoking and Substance Involvement Screening Test) to detect and manage substance-related problems in primary and general medical care settings; the Spanish version of this tool has been adapted and validated [11-13].

The need for other different evaluation means and medical advice to deal with these problems has been highlighted by the users [14]. Computerized versus in-person-based ASSIST interventions have already proven effectiveness in reducing drug consumption in English-speaking samples but not in Spanish samples [15]. Tools that use the internet as an efficient communications channel could be an alternative in dealing with this type of problem, as 78.7% of Spanish families have an internet connection and 64.3% of the population uses it on a daily basis. The current trend is for the internet to be used from handheld mobile devices such as mobile phone [16] instead of personal computers. The advantages of this means for screening include accessibility, 24-hour availability, ease of transfer to users who speak the same language, and the low cost of updating the tools without the need for a major reinvestment in resources. [17].

Screening based on internet platforms and portals has already proved to be useful in other areas, such as mental health [18-20], in relation to high-risk sexual conduct [21], and addictions to alcohol and smoking [22-24]. Some of these have been translated and validated into Spanish. To our knowledge, no Web-based version of this test in Spanish has ever been used for screening [17,25].

ASSIST is designed to detect people at risk of the most common substances used by the general population or in primary care settings. The administration of the ASSIST, by virtue of being a paper-and-pencil assessment requires a great amount of time to be administered in-person in case of subjects complaining about several drugs. Self-administration of the Web-based test could reduce the time required to get the screening done [26].

As part of the Ministry of Health's National Anti-Drug Abuse Plan, the ASSIST website [27] was launched in Madrid on February 2015 as a Web-based self-screening tool for all kinds of substances and brief self-applied intervention for the Spanish-speaking general public, based on the guidelines of the WHO's ASSIST test [28,29].

First, the main objective of this study was to describe the user type for the tool, as well as the drug consumption patterns 6 months after it has been launched. Second, factors associated with the severity of substance use as well as polyconsumption were analyzed.

## Methods

### Design: Cross-Sectional Study

#### Recruitment Methods

To enable access, coverage, and anonymity, it was decided that the screening would take place on a website. The subjects screened from February 24, 2015 to August 24, 2015 (first 6 months) were included in the analysis. It was an open survey, since access was possible as many times as considered necessary without registering. To control duplicate reporting, a multicomponent checkup was developed ahead with cookies used for identification purposes, and a second checkup was applied during analysis. From a promotion and population scope perspective, no initial contact was made with the potential participants on the internet. The tool was publicly launched in the Government Delegation's National Anti-Drug Abuse Agency on February 24, 2015 and promoted by press releases and coverage during the presentation [28,29] without using any other Web-based or printed commercial or publicity campaign. Website was fully dedicated to the screening. A banner in the Agency home page website was placed for the first 3 months. The press release took place on several official websites, platforms, and Spanish medical websites [30-33].

#### Study Population

##### Eligibility Criteria

Subjects who claimed to be older than 18 years when accessing the website, who answered all the ASSIST test questions at one time, and whom we identified as coming from a sole device with a sole identifier (internet protocol, IP) were included, although no IP check was done before the analysis. A screen-printed research informed consent was claimed, which had to be read and validated to ensure acknowledgment before entering the test. No incentives (monetary, prizes, or others) were offered.

##### Web Design and Survey Administration

The entire website [27] is a voluntary Web-based self-screening tool in Spanish that is anonymous and free of charge for the evaluation of drug consumption by subjects older than 18 years (Multimedia Appendix 1). The website was an e-survey itself and was developed in conjunction with a group of IT professionals experienced in health sciences. The site was designed to be displayed both on mobile and desktop viewports. The study does not include the collection of health information with identifiers since it was a blind survey. However, on access to the platform, users were notified that personal data will not be required and that the website will never replace the judgment of a health professional. Users granted consent by clicking that pop-up message. The previous pop-up message plus the 3 initial questions (age, weight, and gender) limited access to automated robots. Users later separately accessed each ASSIST question for each drug. No randomization of ASSIST questionnaires was done to comply with original validated version. All the questions were presented for each drug on 3 different screens (Multimedia Appendix 2). The screening tool performed an automated form validation to ensure that all questions were answered.

After completing the test, the users received a report with a summary of the test results, a comparison with the EDADES [3] survey data, personalized according to the same age range, as well as the digital equivalent of the recommendation and drug-related risk cards for each drug under the Spanish version of ASSIST published by the WHO [12]. This document was available for downloading or printing. Furthermore, if the pattern indicated that intervention was recommended, a space was offered to consult a map of addiction treatment centers available in Spain, according to post codes (Multimedia Appendix 3).

### Measuring Instruments

#### Variables

The main result variable was the score obtained from the answers to the self-administered ASSIST test for each of the screened drugs. ASSIST is a brief questionnaire used to identify risky drug use developed by the WHO and adapted and validated in Spanish. The questionnaire consists of 8 questions on recent and lifelong consumption of 9 substances (tobacco, alcohol, cannabis, cocaine, amphetamines or other stimulants, sleeping pills, hallucinogenic drugs, inhalants, and others). Several domains of the questions are considered (time of use, recent use, desire to consume, health issues, social issues, legal issues, difficulty to stop consuming, etc). According to the WHO, from 0 to 3 points means no intervention is recommended as the risk of a condition related to the substance is low; from 4 to 26 points (11-26 for alcohol), brief intervention is recommended; and for scores of 27 points and above, intensive treatment is recommended. The analysis then classifies the risks into 3 levels (low, moderate, and serious). Other social and demographic variables are recorded such as gender, age, and weight.

#### Sources of Data

Users reported weight, gender, and age anonymously. Self-reported anthropometric data have already proven to be a valid method of collecting these data [34]. The time between clicks was used to estimate the duration of the survey collected automatically. The answers to the ASSIST test questions and the demographic data and resulting scores were stored encrypted.

Google Analytics was used to compare sociodemographic characteristics reported by the users to their Google accounts’ available information. This tool is normally used by Web designers and search-engine-optimization experts to improved usability [35,36], and it gives you the estimated age, gender, locations and preferences of the users accessing your website by executing a JavaScript code when anyone enters the website. Users navigating with an open email account or certain type of Web browsers are giving this demographic information straightaway to this tool according to Google’s privacy terms. This third-party’s policy includes availability of this information to every website designer. The information presented by Google is summarized in central trend and spread measurements. This type of tool has been used in the analysis of similar eHealth environments for guided internet interventions [37] and also uses the website standards established by the Web Analytics Association [38]. Anonymized IP check was done to detect duplicate database entries and last one was kept for analysis.

### Statistical Analysis

The descriptive analysis of the variables was conducted using the central trend and spread measures, if they followed normal distribution and, for asymmetric distributions, medians and interquartile ranges (IQR) were used. In the bivariate analysis, the group averages were compared with the Student *t*-test or the Mann-Whitney *U* test, if the distributions were not normal. In the bivariate analysis of categorical variables, the chi-squared test was used. Uncompleted questionnaires (1.2%) were not analyzed, and atypical timestamp questionnaires (3.4%) were considered faked users and not taken into account for the analysis. Variables with a significant association (*P*<.05) were considered for entry into the logistic regression model. Multiple logistic regression models were performed to determine crude odds ratios, 95% CI, and their corresponding *P* values for each drug. Model fit was assessed using the Hosmer-Lemeshow goodness-of-fit statistic where the model was determined a good fit if the *P* value was not significant.

Stata v14.0 software was used for the log file statistical analysis with *unique* extended commands to identify unique cases (M Hills), *geocode_ip* (S Correia) and *dc* or *Data Check* (JM Domènech) to verify the integrity of the database with unique identifiers. IP, type of device, type of Web explorer, time spent, and demographic data referred were combined to detect unique users.

## Results

### Participants

**Figure 1:**
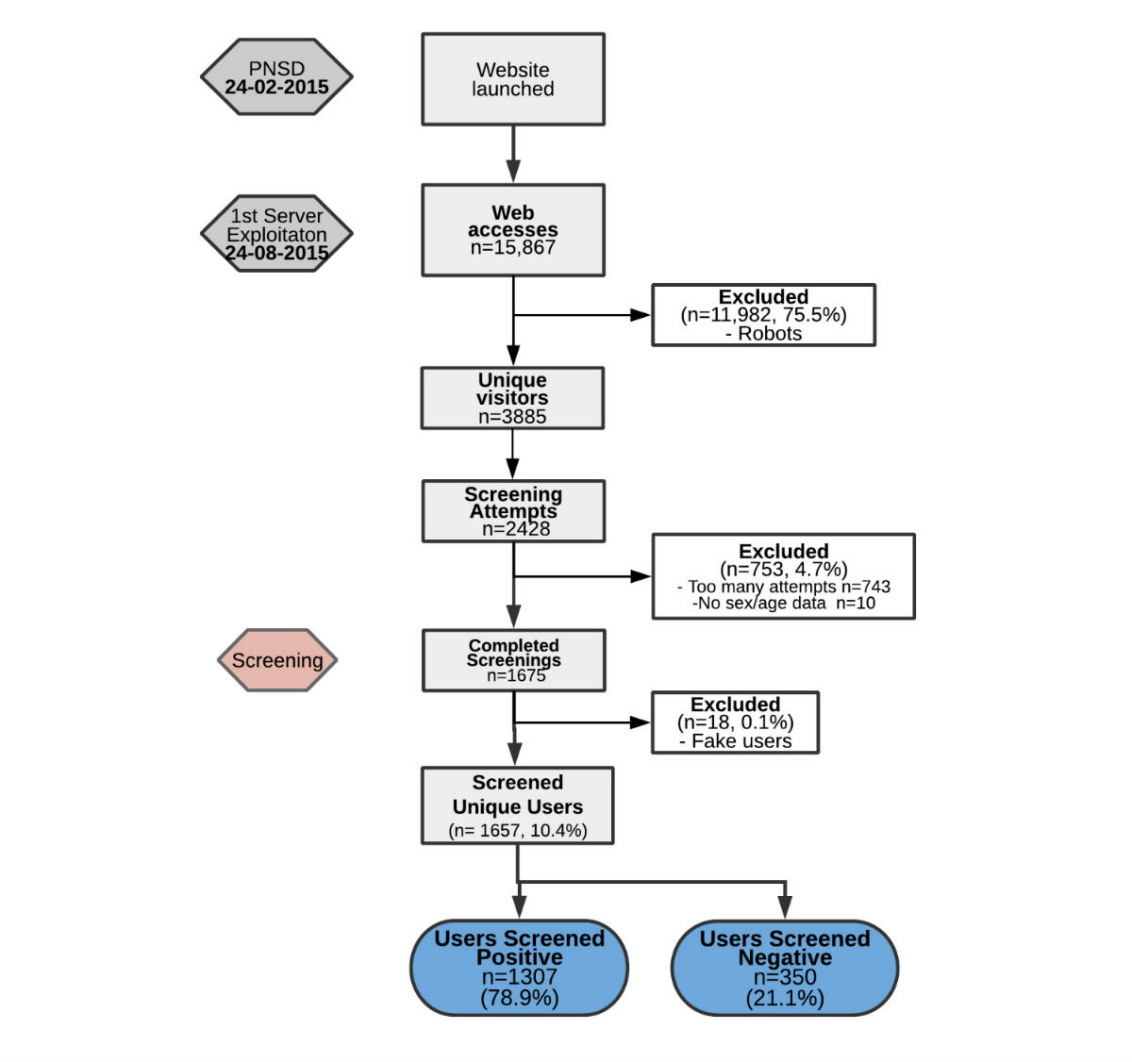
Flow diagram of participants. PNSD: Plan Nacional Sobre Drogas.

From February 24, 2015 to August 24, 2015, the website received a total of 15,867 visits. Figure 1 shows that total unique site visitor as determined by Analytics were 3885, and the test view rate was 2428 (62.50%). A total of 1657 (10.44%) were users who finally completed screening. Participation rate was 1675 of 2242 (74.71%) with a final completion rate of 68.24% (1657/2428).

A total of 76.9% of the access sessions during the period studied took place in the first month after the website was presented, with 3 activity peaks (February 25, launching day, March 4, and March 10, 2015) first one likely related to press release.

**Figure 2:**
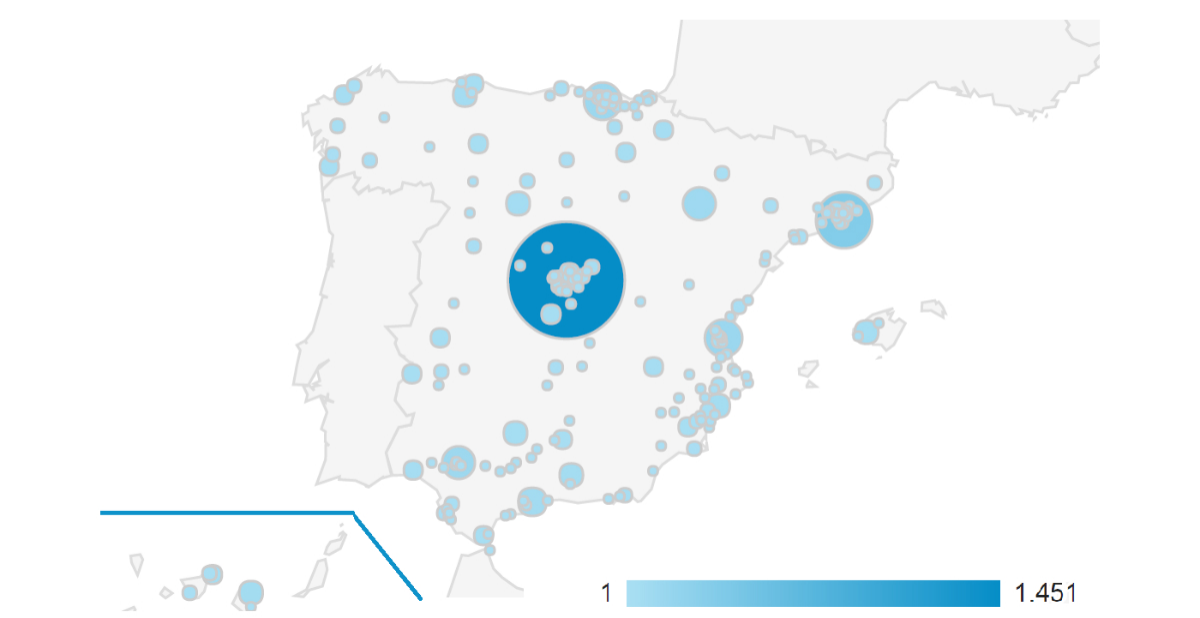
Map of website number of accesses in Spain. The size of the bubble shows the total number of users screened at those locations.

A total of 83.1% of the users were from Spain, 2.3% from Mexico, and 2.2% from Argentina. In Spain, the majority of 35.7% of users were from Madrid, followed by Barcelona (8.2%), Bilbao (3.7%), and Valencia (3.5%, see Figure 2).

Users were mainly from 3 internet sources: 40.2% direct traffic (manual access to the website), 33.2% were redirected from another website related to press release, and 20.3% came from a search engine. A total of 6.4% came from social networks. A multilevel regression model was carried out to analyze the possible influence of location clustering in developing any drug risk showing no significant differences (intraclass correlation coefficient, ICC=.01, 95% CI 0.001-0.11).

**Table 1:** Demographic characteristics referred to by visitors completing the Alcohol, Smoking and Substance Involvement Screening Test (ASSIST).

Characteristics		Total population (n=1657)	Patients screened positive (n=1307)	Patients screened negative (n=350)	*P* value
Gender, men, n (%)		990 (59.74)	812 (62.12)	178 (50.9)	<.001
Age in years (mean, SD)		37.5 (12.9)	37.7 (13.1)	36.9 (12.2)	.30
Weight (median, IQR^a^)		70.9 (60.0-81.0)	71 (22)	71 (20)	.45^b^
Number of drugs reported (median, IQR)		3 (2-4)	2 (1-3)	3 (2-5)	<.001^b^
Duration of screening, minutes (median, IQR)		3 (2-4)	3 (2-4)	2 (1.3-3.0)	<.001^b^
**Month in which screening took place, n (%)**					
	February	526 (32.74)	417 (25.17)	109 (6.8)	
	March	897 (54.13)	700 (43.51)	17 (12.2)	
	April	118 (7.12)	94 (5.79)	24 (1.5)	
	May	40 (2.41)	28 (1.68)	12 (0.8)	
	June	11 (0.66)	8 (0.45)	3 (0.2)	
	July	10 (0.61)	8 (0.45)	2 (0.1)	
	August	8 (0.51)	6 (0.41)	2 (0.1)	
**Type of device, n (%)**					
	Personal computer	1055 (63.66)	810 (48.89)	245 (14.8)	.006
	Phone/tablet	602 (36.31)	497 (30.00)	105 (6.3)	.006
**Referral websites, n (%)**					
	Direct access	743 (44.84)	586 (35.44)	157 (9.4)	.03
	Google search	288 (17.38)	228 (13.78)	60 (3.6)	.03
**Drug information websites, nonprofit**		133 (8.03)	110 (6.53)	23 (1.5)	.03
	Government websites	103 (6.21)	82 (5.01)	21 (1.2)	.03
	Social media	51 (3.07)	41 (2.47)	10 (0.6)	.03

^a^IQR: interquartile range.

^b^Mann-Whitney *U* test.

**Table 2:** Distribution of risk patterns according to the drug and gender. Statistically significant values are in italics.

Drug risks		Total (n=1657), n (%)	Men (n=990), n (%)	Women (n=667), n (%)	*P* value
**Tobacco**					.41
	Not stated	0 (0.00)	0 (0.0)	0 (0.0)	
	Low	817 (49.30)	485 (29.3)	332 (20.0)	
	Moderate	764 (46.10)	454 (27.4)	310 (18.7)	
	Serious	76 (4.58)	51 (3.1)	25 (1.5)	
**Alcohol**					. *001*
	Not stated	200 (12.07)	118 (7.1)	82 (5.0)	
	Low	1020 (61.55)	580 (35)	440 (26.6)	
	Moderate	326 (19.67)	209 (12.6)	117 (7.1)	
	Serious	111 (6.69)	83 (5.0)	28 (1.7)	
**Cannabis**					*<.001*
	Not stated	761 (45.92)	417 (25.1)	344 (20.8)	
	Low	535 (32.28)	310 (18.7)	225 (13.6)	
	Moderate	274 (16.53)	198 (12)	76 (4.5)	
	Serious	87 (5.25)	65 (3.9)	22 (1.2)	
**Cocaine**					*<.001*
	Not stated	1166 (70.36)	632 (38.1)	534 (32.3)	
	Low	408 (24.62)	300 (18.1)	108 (6.5)	
	Moderate	65 (3.92)	47 (2.8)	18 (1.1)	
	Serious	18 (1.08)	11 (0.7)	7 (0.4)	
**Stimulants**					^a^
	Not stated	1316 (79.42)	747 (45.1)	569 (34.3)	
	Low	316 (19.07)	225 (13.6)	91 (5.5)	
	Moderate	18 (1.08)	14 (0.8)	4 (0.2)	
	Serious	7 (0.42)	4 (0.2)	3 (0.2)	
**Inhalants**					^a^
	Not stated	1574 (94.99)	932 (56.3)	642 (38.7)	
	Low	78 (4.71)	53 (3.2)	25 (1.5)	
	Moderate	5 (0.30)	5 (0.3)	0 (0.0)	
	Serious	—	—	—	
**Sedatives/Hypnotics**					*.01*
	Not stated	1190 (71.82)	741 (44.7)	449 (27.1)	
	Low	275 (16.60)	145 (8.7)	130 (7.9)	
	Moderate	162 (9.78)	88 (5.3)	74 (4.5)	
	Serious	30 (1.81)	16 (1)	14 (0.8)	
**Hallucinogenic drugs**					^a^
	Not stated	1423 (85.88)	820 (49.5)	603 (36.4)	
	Low	233 (14.06)	169 (10.2)	64 (3.9)	
	Moderate	1 (0.06)	1 (0.1)	0 (0.0)	
	Serious	—	—	—	
**Opium**					*.019*
	Not stated	1552 (93.66)	918 (55.4)	634 (38.3)	
	Low	91 (5.49)	65 (3.9)	26 (1.6)	
	Moderate	11 (0.66)	7 (0.5)	4 (0.2)	
	Serious	3 (0.18)	0 (0)	3 (0.2)	

^a^n<5=less than 5 cases.

### Consumption Patterns

The average age of the subjects was 37.4 years (standard deviation, SD 12.9) and 59.9% of cases stated to be male. Users could test themselves for drug-related risks and no risks could be showed. Of all the users screened, 21.1% did not have any moderate to high substance-related risks (as opposed to 78.9% with a moderate or high risk in relation to at least 1 substance). The average time spent taking the test was 3 min (IQR: 2-4), with a statistically significant higher time spent for those who screened positive on the test for at least 1 drug. The number of drugs reported was higher for the group who screened positive compared with the group who showed no moderate to high drug-related risks (*P*<.001); see Table 1.

The most highly consumed drug by men as well as women was tobacco, which all the self-surveyed subjects stated to smoke at some point, followed by alcohol (87.9%) and cannabis (54.1%). We found moderate or serious substance-related risk for tobacco (50.7%), alcohol (26.4%), cannabis (21.8%), and sedatives or hypnotics (11.6%) as the 4 most frequent.

In the subgroup analysis, significant differences were observed based on gender: men had moderate to high risks for alcohol disorders in 17.6% of cases compared with women in 8.8% (*P*=.001) of cases; in the consumption of cannabis, men had 15.9% cases compared with women in 5.9% (*P*<.001) cases, and in the consumption of cocaine, men had 3.5% of cases compared with women in 1.5% (*P*<.001) cases (see Table 2). Male respondents had 1.55 times risk of having alcohol problems (odds ratio, OR 1.55, 95% CI 1.18-2.04; *P*=.002) and had double the risk for cannabis problems (OR 2.07, 95% CI 1.46-2.92; *P*<.001); see Table 3.

In the subgroup analysis of age categories, users who screened positive for moderate to high substance-related risks were distributed into groups up to 44 years of age. The highest number of users with drug-related risks was in the 35- to 44-year-old category: tobacco (12.3% moderate, 1.6% serious), alcohol (5.3% moderate; 1.9% serious), cocaine (1.6% moderate), and sedatives/hypnotics (2.6% moderate). The cannabis-related risks were concentrated in the 2 youngest age groups (18-24 and 25-34 years) with 5.7% of all subjects consuming the drug. Growing age increased 3 times risk of developing alcohol problems for people between 45 and 65 years (OR=3.01, 95% CI 1.89-4.79; *P*<.001). In contrast, increasing age protects against developing cannabis-related problems (OR=0.30, 95% CI 0.17-0.56; *P*<.001); see Table 3.

With regard to polyconsumption or the consumption of different drugs throughout the same period of time, a moderate or serious risk was observed in 31.6% of the subjects. Number of substances and sex distribution are shown in Table 4. Male respondents, compared with female respondents, were more likely to develop polyconsumption (OR=1.92, 95% CI 1.5-2.47; *P*<.001) *.* Those user screening by mobile devices had higher risk of developing problems even when adjusting for potential confounder such as age (OR=1.41, 95% CI 1.1-1.8, *P*=.006) *.* Age showed no significant differences regarding poly-consumption.

Table 5 shows the combinations of the most common moderate or serious drugs-related risks for 2 drugs and the percentage of polyconsumers of each pattern. The most frequent combinations found were tobacco and cannabis (31.3%), tobacco and alcohol (16.2%), and tobacco and sedatives (8.4%). There was also a group of subjects with a serious risk for each such as tobacco and cannabis serious (10.4%) and tobacco and alcohol serious (7.2%).

**Table 3:** Factors associated with moderate to high risky drug use estimates in general people screened at the website. Statistically significant values are in italics.

Sociodemographic factors		Tobacco (n=840)		Alcohol (n=437)		Cannabis (n=361)	
		OR (95% CI)	*P* value	OR (95% CI)	*P* value	OR (95% CI)	*P* value
**Sex**							
	Female	1.00 (Ref)		1.00 (Ref)		1.00 (Ref)	
	Male	1.21 (0.96-1.51)	.10	1.55 (1.18-2.04)	*.002*	2.07 (1.46-2.92)	*<.001*
**Age group (years)**							
	18-24	1.00 (Ref)		1.00 (Ref)		1.00 (Ref)	
	25-34	0.74 (0.53-1.03)	.08	1.39 (0.89-2.15)	.15	0.56 (0.38-0.84)	*.005*
	35-44	0.81 (0.58-1.15)	.24	1.83 (1.18-2.84)	*.007*	0.34 (0.20-0.53)	*<.001*
	45-54	0.83 (0.57-1.21)	.34	3.01 (1.89-4.79)	*<.001*	0.30 (0.17-0.56)	*<.001*
	55-64	0.92 (0.59-1.43)	.70	3.08 (1.79-5.31)	*<.001*	0.14 (0.05-0.45)	*.001*
	>65	0.84 (0.43-1.64)	.60	3.73 (1.69-8.21)	*.001*	—	
**Accessing device**							
	Personal computer/mac	1.00 (Ref)		1.00 (Ref)		1.00 (Ref)	
	Mobile phone/tablet	1.47 (1.16-1.86)	*.001*	0.80 (0.61-1.07)	.14	1.48 (1.06-2.06)	*.021*
	Website referral source						
	None	1.00 (Ref)		1.00 (Ref)		1.00 (Ref)	
	Official press releases	0.98 (0.64-1.50)	.91	0.96 (0.57-1.63)	.89	1.48 (0.60-1.34)	.20
	Google searches	1.08 (0.82-1.41)	.58	1.06 (0.76-1.47)	.74	0.90 (0.60-1.34)	.6
	Drug information websites	1.81 (1.20-2.72)	*.005*	1.62 (1.02-2.56)	*.039*	0.47 (0.27-0.84)	*.011*
	Social media referrals	1.27 (0.71-2.26)	.42	1.38 (0.71-2.71)	.35	0.84 (0.60-1.34)	.68
	Time spent in the test	1.47 (1.16-1.86)	*.001*	1.08 (1.04-1.12)	*<.001*	1.08 (1.02-1.14)	*.006*

**Table 4:** Gender differences and number of substance-related risks at screening.

Number of substances at risk	Total (n=1657, n (%)	Men (n=990), n (%)	Women (n=657), n (%)	*P* value
No substance	350 (21.12)	178 (17.9)	172 (25.8)	
1 substance	780 (47.07)	452 (45.7)	328 (49.2)	*.026*
2 substances	431 (26.01)	295 (29.8)	136 (20.4)	*<.001*
3 substances	77 (4.64)	55 (5.6)	22 (3.3)	*.001*
4 substances	13 (0.78)	7 (0.7)	6 (0.9)	*.83*
5 or more substances	6 (0.36)	3 (0.3)	3 (0.5)	^a^

^a^n<5=less than 5 cases.

**Table 5:** Qualitative description of polyconsumers of 2 substances (n=431).

Drugs patterns		Tobacco n (%)		Alcohol n (%)		Cannabis n (%)		Cocaine n (%)		Amphetamine n (%)		Inhalants n (%)		Sedatives n (%)		Opium n (%)	
		Mod^a^	Ser^b^	Mod	Ser	Mod	Ser	Mod	Ser	Mod	Ser	Mod	Ser	Mod	Ser	Mod	Ser
**Tobacco**																	
	Mod																
	Ser																
**Alcohol**																	
	Mod	70 (16.2)	—														
	Ser	31 (7.2)	5 (1.2)	`													
**Cannabis**																	
	Mod	135 (31.3)	8 (1.9)	8 (1.9)	1 (0.2)												
	Ser	45 (10.4)	8 (1.9)	—	—												
**Cocaine**																	
	Mod	13 (3.0)	2 (0.5)	7 (1.6)	1 (0.2)	3 (0.7)	1 (0.2)										
	Ser	1 (0.2)	1 (0.2)	—	—	1 (0.2)	1 (0.2)										
**Amphetamine**																	
	Mod	2 (0.5)	—	2 (0.5)	—	2 (0.5)	—	1 (0.2)	—								
	Ser	2 (0.5)	—	—	—	—	—	—	1 (0.2)								
**Inhalants**																	
	Mod	2 (0.5)	—	—	—	1 (0.2)	—	—	—	—	—						
	Ser	—	—	—	—	—	—	—	—	—	—						
**Sedatives**																	
	Mod	36 (8.4)	3 (0.7)	15 (3.5)	5 (1.2)	1 (0.2)	1 (0.2)	1 (0.2)	—	—	—	—	—				
	Ser	3 (0.7)	2 (0.5)	2 (0.5)	3 (0.7)	—	—	—	—	—	—	—	—				
**Opium**																	
	Mod	2 (0.5)	—	—	—	—	—	—	—	—	—	—	—	1 (0.2)	—		
	Ser	—	—	—	—	—	—	—	—	—	—	—	—	—	—		

^a^Mod: moderate.

^b^Ser: serious.

^c^Hallucinogenic drugs were not included, as there were no positive screening results of polyconsumption (2 or more).

## Discussion

### Principal Findings

In the first 6 months after the Web-based self-screening for drug consumption website was launched, a total of 15,867 users were recorded, of which 1657 (10.4%) completed the screening. The average age obtained was 37.4 years and 78.9% showed moderate or serious drug-related risks. The most highly consumed drugs stated were tobacco (50.7%), alcohol (26.4%), cannabis (21.8%), and sedatives (11.6%) with men taking more alcohol, cannabis, or cocaine; and young people (aged 18-35 years) using more cannabis. Polyconsumption was observed in 31.6% of the cases.

In terms of the number of users, our website showed data similar to others in the existing literature [17]. Nevertheless, it is true that other single-drug platforms, such as those for only alcohol screening, have achieved a greater scope with larger samples after targeted marketing campaigns also on the internet, as well as by mailings and massive letter box drops of pamphlets [23,24].

Our website user map (see Figure 2) shows that the geographic distribution of users with drug problems are similar to that found in the literature [3], the most common cities still being Madrid and coastal cities (Barcelona, Valencia, and Bilbao). This could be explained either because big cities have higher drug use rates [3] or just because they are major cities and we are getting more respondents from these cities. However, after adjusting in a multilevel regression model, cluster had no influence on developing drug problems.

Users surfing the Web using Google services are giving their demographic characteristics and interest information for analysis according to the Google privacy terms. A total of 52.1% of users could be analyzed, and differences were observed in relation to age and gender. In the data provided by the users, there were 59.9% men, as opposed to 41.2% in the Google sample, and 7.1% of users stated in the test that they were younger (18-24) than when compared with the data obtained with Google. This could be due to the 50% of incomplete information from the Google remaining sample, or if representation is assumed, due to the fact that some people did the test for others.

Drug-use patterns partially differ with respect to consumption prevalence in Spain according to the EDADES [3], in which hypnotics-sedatives were higher than cannabis in the 15- to 64-year-old population and, in our sample, cannabis was still the third highest consumed drug. The characteristics of both samples could explain this difference, given that the upper age ranges are more highly represented in the EDADES study, as opposed to our sample and, in addition, because these are drugs that are initially consumed at middle age. Furthermore, both tests differ since the EDADES questionnaire addresses mostly consumption rates but does not address other type of drug-related risks for nondependent people.

In terms of polyconsumption, there are also differences: the definition used in the EDADES survey refers to the combined consumption of substances in the same period [3]. The ASSIST test questions say, “at some time” and “in the last three months,” therefore, to a certain extent, it already partly includes that information. Moreover, the ASSIST offers an estimation of the clinical and social risks related to substance use. Therefore, as far as terminology is concerned, our polyconsumption is the combination of moderate and serious risk estimates relating to any of 2 or more substances detected. This is clinically relevant as the users are at a greater risk and may need more intense treatment.

### Applicability of the Results

This is the first drug-related risks screening website related to all common drugs, using a scale that has been internationally validated by the WHO (ASSIST), adapted to Spanish for Web-based screening.

The results also show a target group for potential intervention relating to the tobacco-cannabis polyconsumption pattern, due to the high percentage detected in certain age groups (18-34 years).

One of the major advantages of this type of tool would be the possibility of reaching populations that would otherwise delay resorting to the health system and also offering primary care doctors and other health care professionals a valid tool for handling the risks related to the consumption of substances before it becomes an established medical condition.

The potential impact of applying overall prevention measurements (screening as secondary prevention), instead of those addressed to specific populations is a public health issue that is currently being debated. The screening in itself would be pointless if counteraction was not offered. There is already evidence of decreasing mortality rates in favor of global intervention after screening general population samples by models designed for cardiovascular disease, as opposed to screening specific population alone [39]. Drug-related interventions such as brief interventions using ASSIST Web-based tools have already proven effectiveness in the English samples compared with in-person brief interventions [15].

### Limitations

One of the main limitations would be the ability to identify sole users and therefore to eliminate duplicating or overestimating the results. Several users could have access from the same device and even from the same network to complete the test, without being able to make a distinction between them. Therefore, the server data were exploited, limited by the anonymized IP address, compared with the data from the device used for access and the time (day and time) that the test was taken and, together with the aforementioned demographic data, an algorithm was established to identify single and valid users. When the users could not be differentiated, an attempt was made to eliminate “testing users” from the analysis to identify erroneous situations such as the screening of many drugs in a short period of time, several attempts by the same user, etc. For users making several attempts, the last answer was accepted as valid.

Another limitation that can be assumed is related to the scope of publication. Users accessed the website mainly because they had heard about it or were redirected by a different website; 1 of every 5 did so after browsing with Google. Given that the environment was created under a research framework publicized by a public body (National Anti-Drug Abuse Plan), access and users came from locations where there was publicity resulting from the presentation. Future studies would benefit by including a broader population to improve external validity.

Another difficulty encountered was the restriction of the screening age to 18 years and older. At the time we developed the website, validation for the adolescent population had not been published. In 2015, Gryczynksi et al [40] validated this tool for adolescents in primary health care, meaning that it could therefore be extrapolated to our general population. Its adaptation to our environment would be simple, as it requires minimal programming changes to allow access to younger users (12-17 years) according to the aforementioned validation.

## Appendix

Multimedia Appendix 1 Home page.

Multimedia Appendix 2 Drug screening page.

Multimedia Appendix 3 Drug risks evaluation webpage.

## Abbreviations

ASSIST: Alcohol, Smoking and Substance Involvement Screening Test
IP: internet protocol
OR: odds ratio
PNSD: Plan Nacional Sobre Drogas
WHO: World Health Organization
