# Supplementary figures and images for: Web-Based Alcohol, Smoking, and Substance Involvement Screening Test Results for the General Spanish Population: Cross-Sectional Study
Source: J Med Internet Res. 2018 Feb 16;20(2):e57. doi: 10.2196/jmir.7121 (PMC5834753; doi:10.2196/jmir.7121)

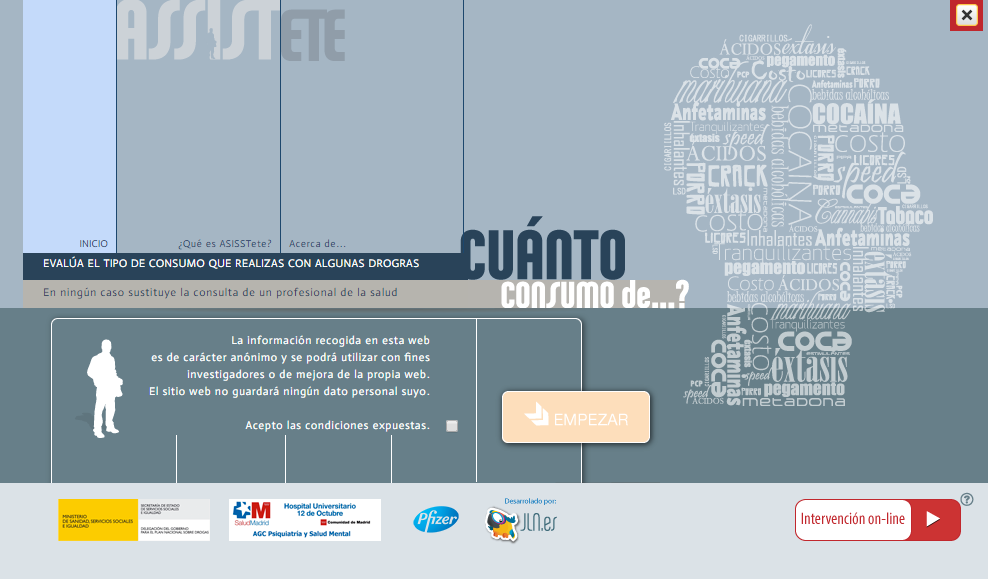

Supplement: Multimedia Appendix 1 [file jmir_v20i2e57_app1.PNG]

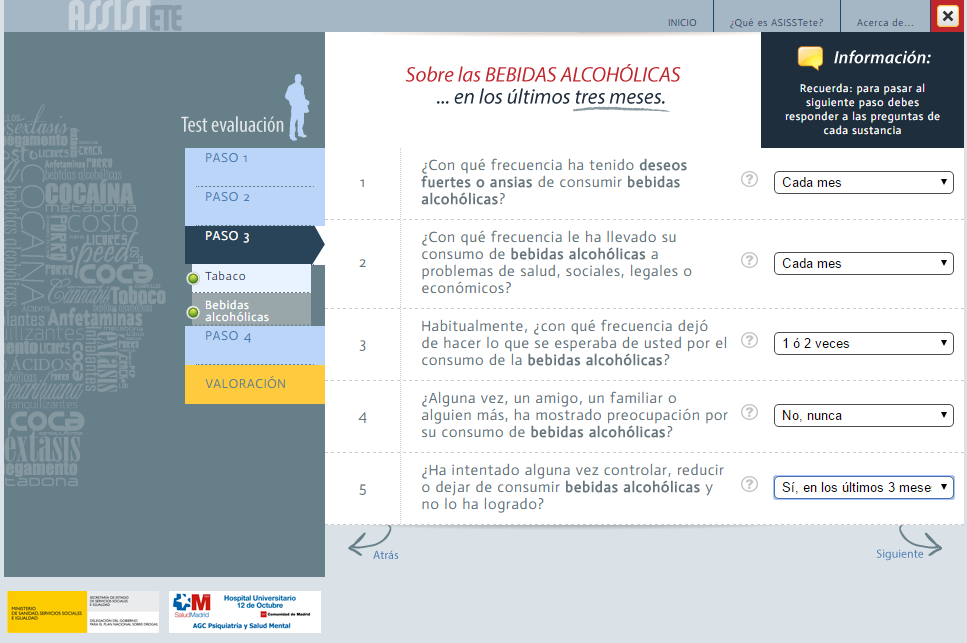

Supplement: Multimedia Appendix 2 [file jmir_v20i2e57_app2.PNG]

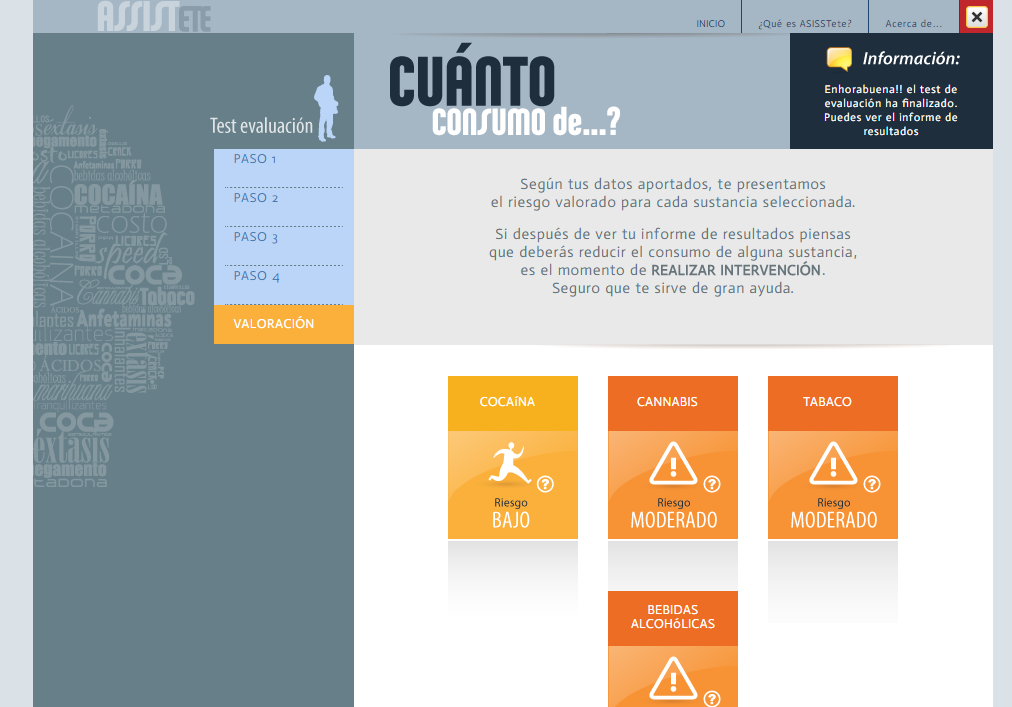

Supplement: Multimedia Appendix 3 [file jmir_v20i2e57_app3.PNG]
